# The difference in hematocrit and albumin levels and the risk of sepsis for patients with acute pancreatitis: a retrospective cohort study based on the MIMIC-IV database

**DOI:** 10.1590/1516-3180.2024.0401.14042025

**Published:** 2025-11-21

**Authors:** Mingjie Jin, Yanmin Wu, Bin Ye

**Affiliations:** I Physician, Department of Ultrasonography, The First Affiliated Hospital, Wenzhou Medical University, Wenzhou, Zhejiang, China.; II Physician, Department of Ultrasonography, Yueqing People’s Hospital, Wenzhou, Zhejiang, China.; III Physician, Department of Ultrasonography, Wenzhou TCM Hospital of Zhejiang Chinese Medical University, Wenzhou, Zhejiang, China.

**Keywords:** Hematocrit, Albumin, Sepsis, Pancreatitis, Mortality, Hematocrit-albumin, Acute pancreatitis, Sepsis, Association

## Abstract

**BACKGROUND:**

Acute pancreatitis (AP) is a major cause of gastrointestinal hospitalization, with an annual global incidence of 3.07%. Severe AP develops in up to 20% of cases, with sepsis occurring in 40–70% of such cases, leading to higher mortality. The early detection of sepsis is crucial. Hematocrit (HCT) and albumin (ALB) levels are individually linked to sepsis. Their combined measure, HCT-ALB, indicates blood and nutritional health. HCT-ALB can predict sepsis and infection outcomes; however, its effectiveness in treating AP-related sepsis has not been investigated.

**OBJECTIVE:**

This study aimed to examine the association between HCT-ALB values and sepsis risk in patients with AP.

**METHODS:**

This retrospective cohort study used Medical Information Market for Intensive Care IV database data. The primary outcome was the risk of sepsis in patients with AP. The HCT-ALB value refers to the difference between HCT and ALB levels, which we categorized into three groups according to quantiles: < 0.5, 0.5–7.6, and ≥ 7.6. Logistic regression models were used to assess the association between HCT-ALB values and sepsis. The predictive value of HCT-ALB was assessed using a receiver operating characteristic curve. Subgroup analyses were conducted for different subgroups.

**RESULTS:**

Among 565 patients with AP, 163 developed sepsis. In the multivariable model, HCT-ALB ≥ 7.60 was associated with sepsis risk for patients with AP [odds ratio (OR) 1.82, 95% confidence interval (CI) 1.06–3.14]. The area under the curve (AUC) value of HCT-ALB in predicting sepsis risk among patients with AP was 0.599 (95% CI 0.544–0.654), which was higher than that of the bedside index for severity in acute pancreatitis score (AUC 0.558, 95% CI 0.509–0.607). Subgroup analysis showed that HCT-ALB was only related to sepsis risk in male patients with acute kidney injury and Sequential Organ Failure Assessment in < 2 subgroups.

**CONCLUSION:**

HCT-ALB values ≥ 7.6 were associated with increased sepsis risk in patients with AP. HCTALB may contribute to identifying the risk of sepsis in patients with AP.

## INTRODUCTION

 Acute pancreatitis (AP) is considered the primary reason for hospital admissions related to gastrointestinal disorders in the United States and in several other countries.^1^ A meta-analysis assessing trends in the global incidence of AP highlighted an annual rise in the overall incidence rate of AP of 3.07%, placing an increasing burden on healthcare systems.^2^ Approximately 15–20% of patients with AP may experience exacerbation, resulting in the development of systemic inflammatory response syndrome (SIRS) and multiple organ failure. Ultimately, this progression can culminate in severe AP.^3^ Sepsis is a life-threatening SIRS resulting from an uncontrolled immune reaction to infection, leading to septic shock and subsequent multiple organ failure.^4^ Secondary pancreatic infection and sepsis, occurring in 40–70% of patients with AP, are associated with elevated mortality rates and a poor prognosis.^3^ Early identification of sepsis risk among such patients is crucial in reducing mortality rates and alleviating the burden of disease. 

 Hematocrit (HCT) levels represent the proportion of red blood cells within the total volume of whole blood, serving as a critical biomarker for diagnosing anemia.^5^ One retrospective cohort study reported that an elevated HCT level was a risk factor associated with the development of postoperative sepsis among older adult patients.^6^ Albumin (ALB) is a pivotal biomarker associated with infection.^7^ In older adult patients with hip fractures, ALB levels < 38 g/L are associated with a higher risk of postoperative infections.^8^ Moreover, ALB levels are used to indicate the severity of sepsis.^9^ HCT-ALB values, which represent the difference between HCT and ALB levels, have recently been used to indicate a patient’s nutritional status.^10-12^ HCT-ALB values have been widely reported as potential biomarkers in several studies. One retrospective study involving data derived from two large databases reported a significant association between elevated HCT-ALB values and an increased risk of intensive care unit (ICU) and hospital mortality among older adult patients with sepsis.^11^ In a retrospective case-control study published in 2020, Dai et al.^12^ reported that elevated HCT-ALB values were observed in patients diagnosed with infectious diseases. Therefore, determining the HCT-ALB value (> 10.25) could potentially be a valuable tool for rapid diagnosis of infectious diseases. However, few studies have investigated the potential value of HCT-ALB in patients with AP. 

 This retrospective cohort study aimed to investigate the association between HCT-ALB values and the risk of sepsis among patients with AP using data derived from the Medical Information Mart for Intensive Care IV (MIMIC-IV) database. 

## MATERIALS AND METHODS

### Data sources

 Data were obtained from the MIMIC-IV database, a large, single-center, free public database containing clinical information in relation to patients at Beth Israel Deaconess Medical Center in Boston between 2008 and 2019.^13^ Patient demographics, admission records, vital signs, laboratory tests, medications, and survival data are recorded in the MIMIC-IV database. Access to this database was approved by the Institutional Review Committee of the Massachusetts Institute of Technology and the Beth Israel Deaconess Medical Center. 

### Study population

**Figure 1:**
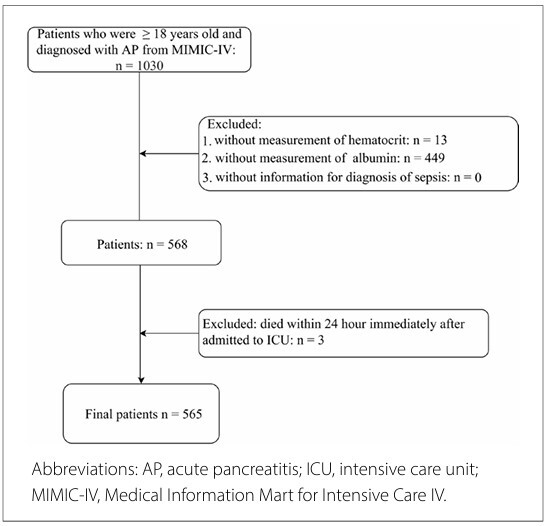
Patient flowchart

 The study inclusion criteria comprised patients aged ≥ 18 years old and diagnosed with AP. Patients with AP were identified using International Classification of Diseases (ICD) (9th revision, code 5770; 10th revision, code K85) codes.^14^ The exclusion criteria comprised patients with missing information regarding HCT or ALB levels, or those with a diagnosis of sepsis. We also excluded patients who had died within 24 h of admission to the ICU. Finally, 565 patients diagnosed with AP were included in the subsequent analysis (**Figure 1**). 

### Data collection

 The primary outcome was the risk of sepsis in patients with AP. A sepsis diagnosis was made based on Sepsis-3 criteria, which involves patients exhibiting signs of infection and a sudden increase in their sequential organ failure assessment (SOFA) score by at least 2 points.^15^ HCT-ALB values, reflecting the difference between the HCT (%) and ALB (g/L) levels, were categorized into three groups according to quantiles, namely, < 0.5, 0.5–7.6, and ≥ 7.6. 

 In the PACU, patients experiencing moderate to severe pain (defined as a numeric rating scale [NRS] score > 3) received intravenous morphine: 0.03 mg/kg for moderate pain and 0.05 mg/kg for severe pain. The goal was to reduce pain to an NRS score of 3 or lower (0, no pain; 10, worst pain imaginable). Patients presenting with PONV were treated with 30 mg of intravenous dimenhydrinate. In the ward, all patients followed the institutional protocol and received intravenous metamizole (2 g every 6 h), intravenous ketoprofen (100 mg every 12 h), intravenous ondansetron (4 mg as needed for nausea), and intravenous tramadol (100 mg every 6 h if NRS > 3). Patients who required tramadol were reassessed 30 min after administration. For patients with moderate-to-severe pain after administration of metamizole, ketoprofen, and tramadol, a medical evaluation was requested, and intravenous morphine (2 mg) was administered every 15 min until mild pain was achieved. 

 Possible confounding variables were included in this analysis, as follows: age (years); sex; ethnicity; marital status; mean blood pressure (MBP, mmHg); respiratory rate; temperature (^°^C); creatinine (mg/dL), hemoglobin (g/dL), blood urea nitrogen (BUN, mg/dL), and platelet (K/uL) levels; red blood cell distribution width (RDW, %); chloride (mEq/L), bilirubin (mg/dL), sodium (mEq/L), bicarbonate (mEq/L), potassium (mEq/L), phosphate (mg/dL), alanine aminotransferase (ALT, IU/L), glucose (mg/dL), aspartate aminotransferase (AST, IU/L), and oxygen saturation (SpO_2_, %) values; prothrombin time (PT, seconds); the international normalized ratio (INR, %); lipase (U/L) levels; the bedside index for severity in acute pancreatitis (BISAP), SOFA, and Glasgow Coma Scale (GCS) scores; the Charlson comorbidity index (CCI); the diagnosis of heart failure, hypertension, diabetes mellitus, renal failure, liver disease, acute kidney injury (AKI), and chronic obstructive pulmonary disease (COPD); and the use of antibiotic treatment, renal replacement therapy (RRT), mechanical ventilation, vasopressors, and enteral nutrition. For patients with multiple IUC admissions, only data in relation to their initial ICU admissions were analyzed. 

### Ethics approval and consent to participate

 MIMIC-IV is a public database, and ethical approval was obtained concerning the use of data in relation to patients registered in the database. Users can download relevant data free of research fees and publish relevant articles. 

### Statistical analysis

 Continuous variables following normal distribution are described using mean and standard deviation (SD, mean ± SD), and t-tests were conducted for group comparisons. Non-normal data are presented as median and interquartile spacing (M [Q1, Q3]), and group comparisons were analyzed using a Mann–Whitney U rank sum test. Categorical variables are reported as the number of cases and composition ratio (n [%]), and a chi-square test was used for group comparisons. The specified predictive mean matching method was used to interpolate all missing variables, and a sensitivity analysis of the data was performed pre- and post-interpolation (**Appendix 1**). A univariate logistic regression model was used to identify potential confounding variables related to sepsis (**Appendix 2**). Univariate and multivariate logistic regression models with HCT-ALB as the independent variable and sepsis as the outcome variable were used to assess the association between HCT-ALB values and sepsis risk in patients with AP. Odds ratios (ORs) and 95% confidence intervals (95% CIs) were then calculated. Receiver-operating characteristic (ROC) curve analysis was used to assess the predictive value of HCT-ALB. Subgroup analyses were conducted for the following predefined populations: stratified by sex (male/female), by history of AKI (yes/no), and by Sequential Organ Failure Assessment (SOFA) score (< 2 or ≥ 2). The significance threshold was set at P < 0.05, and statistical analyses were conducted using SAS (version 9.4; SAS Institute Inc., Cary, North Carolina) software. 

#### Availability of data and materials

The datasets used and/or analyzed in the current study are available from the corresponding author upon reasonable request.

## RESULTS

### Patient characteristics

 This study included 565 patients with AP (women, n = 246 [43.54%]; men, n = 319 [56.46%]). As shown in Table 1,the average patient age was 57.80 ± 17.61 years, while the median HCTALB value was 4 (range, −1–9.6). Based on the development of sepsis, patients with AP were categorized into two groups, namely, those with sepsis (sepsis, n = 163) and those without (non-sepsis, n = 402), and patient characteristics were compared between the two patient groups (Table 1). The sepsis group had decreased ALB and MBP values and higher HCT-ALB values and SOFA scores than in the non-sepsis group. 

**Table 1:** Baseline characteristics between the non-sepsis and sepsis groups

**Variables**		**Total (n = 565)**	**Groups**		**Statistics**	**P value**
			**Non-sepsis (n = 402)**	**Sepsis (n = 163)**		
Age, years; mean ± SD		57.8 ± 17.61	58.56 ± 18.10	55.91 ± 16.26	t = 1.62	0.105
Sex, n (%)						
	*Women*	246 (43.54)	173 (43.03)	73 (44.79)	χ^2^ = 0.145	0.704
	*Men*	319 (56.46)	229 (56.97)	90 (55.21)		
Ethnicity, n (%)						
	*African American*	55 (9.73)	41 (10.2)	14 (8.59)	χ^2^ = 2.358	0.501
	*European*	348 (61.59)	253 (62.94)	95 (58.28)		
	*Other*	67 (11.86)	44 (10.95)	23 (14.11)
	*Ethnicity not stated*	95 (16.81)	64 (15.92)	31 (19.02)
Marital status, n (%)						
	*Married*	223 (39.47)	161 (40.05)	62 (38.04)	χ^2^ = 3.381	0.496
	*Never married*	178 (31.5)	120 (29.85)	58 (35.58)		
	*Divorced*	43 (7.61)	32 (7.96)	11 (6.75)		
	*Widowed*	53 (9.38)	42 (10.45)	11 (6.75)		
	*Unknown*	68 (12.04)	47 (11.69)	21 (12.88)		
HCT, %; mean ± SD		34.06 ± 6.91	33.96 ± 6.4	34.31 ± 8.04	t = −0.48	0.628
ALB, g/L; mean ± SD		29.78 ± 6.23	30.43 ± 6.16	28.17 ± 6.14	t = 3.97	< 0.001
HCT-ALB, M (Q_1_, Q_3_)		4.00 (−1, 9.6)	3.20 (−1.3, 8.5)	6.3 (0.4, 13.1)	Z = 3.69	< 0.001
HCT-ALB, n (%)						
	*< 0.50*	184 (32.57)	143 (35.57)	41 (25.15)	χ^2^ = 14.115	< 0.001
	*0.50−7.60*	193 (34.16)	144 (35.82)	49 (30.06)		
	*≥ 7.60*	188 (33.27)	115 (28.61)	73 (44.79)		
MBP, mmHg; mean ± SD		91.71 ± 19.17	93.25 ± 18.57	87.9 ± 20.11	t = 3.03	0.003
Respiratory rate, mean ± SD		21.66 ± 6.67	21.6 ± 6.65	21.8 ± 6.75	t = −0.33	0.739
Temperature, °C; mean ± SD		36.84 ± 1	36.79 ± 0.96	36.97 ± 1.09	t = −1.83	0.069
Creatinine, mg/dL; M (Q_1_, Q_3_)		1.1 (0.7, 2)	1 (0.7, 1.6)	1.50 (0.9, 2.8)	Z = 4.417	< 0.001
Hemoglobin, g/dL; mean ± SD		11.28 ± 2.33	11.27 ± 2.2	11.31 ± 2.64	t = −0.18	0.854
BUN, mg/dL; M (Q_1_, Q_3_)		20 (12, 37)	17.5 (11, 35)	26 (17, 44)	Z = 4.797	< 0.001
RDW, %; mean ± SD		15.21 ± 2.18	15.14 ± 1.91	15.38 ± 2.73	t = −0.99	0.323
Platelet, K/uL; M (Q_1_, Q_3_)		182 (125, 265)	183.5 (128, 267)	181 (124, 248)	Z = −0.888	0.375
Bilirubin, mg/dL; M (Q_1_, Q_3_)		1.1 (0.6, 3)	1 (0.6, 3)	1.1 (0.6, 2.9)	Z = 0.726	0.468
Bicarbonate, mEq/L; mean ± SD		20.65 ± 5.66	21.08 ± 5.52	19.58 ± 5.88	t = 2.88	0.004
Sodium, mEq/L; mean ± SD		138.38 ± 5.91	138.3 ± 5.44	138.55 ± 6.93	t = −0.41	0.682
Potassium, mEq/L; mean ± SD		4.16 ± 0.91	4.12 ± 0.92	4.25 ± 0.88	t = −1.52	0.13
Chloride, mEq/L; mean ± SD		104.65 ± 7.33	104.31 ± 7.12	105.49 ± 7.78	t = −1.74	0.083
Phosphate, mg/dL; M (Q_1_, Q_3_)		3.3 (2.3, 4.2)	3.2 (2.2, 4)	3.6 (2.5, 4.9)	Z = 3.231	0.001
Glucose, mg/dL ; M (Q_1_, Q_3_)		124 (100, 174)	121 (99, 167)	137 (104, 194)	Z = 2.425	0.015
ALT, IU/L; M (Q_1_, Q_3_)		54 (25, 168)	54.5 (25, 168)	54 (26, 167)	Z = 0.109	0.913
AST, IU/L; M (Q_1_, Q_3_)		78 (38,187)	72.5 (35,182)	87 (46, 216)	Z = 2.115	0.034
SpO_2_, %; mean ± SD		95.93 ± 4.58	96.01 ± 4.44	95.72 ± 4.93	t = 0.68	0.498
INR, M (Q_1_, Q_3_)		1.3 (1.1, 1.6)	1.3 (1.1, 1.6)	1.4 (1.2, 1.7)	Z = 2.295	0.022
PT, seconds; M (Q_1_, Q_3_)		14.4 (13, 17.3)	14.2 (13, 17)	14.9 (13.2, 17.9)	Z = 1.839	0.066
Lipase, n (%)					
	*< 208 U/L*	158 (27.96)	108 (26.87)	50 (30.67)	χ^2^ = 0.863	0.65
	*≥ 208 U/L*	217 (38.41)	156 (38.81)	61 (37.42)		
	*Unknown*	190 (33.63)	138 (34.33)	52 (31.9)		
SOFA, M (Q_1_, Q_3_)		2 (0, 5)	2 (0, 4)	4 (1, 7)	Z = 6.027	< 0.001
BISAP, M (Q_1_, Q_3_)		2 (1, 3)	2 (1, 3)	2 (1, 3)	Z = 2.28	0.023
GCS, mean ± SD		14.53 ± 1.58	14.56 ± 1.43	14.45 ± 1.91	t = 0.69	0.491
CCI, M (Q_1_, Q_3_)		2 (1, 3)	2 (1, 3)	2 (1, 4)	Z = 0.188	0.851
Heart failure, yes; n (%)		83 (14.69)	58 (14.43)	25 (15.34)	χ_2_ = 0.077	0.782
Hypertension, yes; n (%)		321 (56.81)	234 (58.21)	87 (53.37)	χ_2_ = 1.105	0.293
Diabetes, yes; n (%)		170 (30.09)	127 (31.59)	43 (26.38)	χ_2_ = 1.498	0.221
Renal failure, yes; n (%)		61 (10.8)	37 (9.2)	24 (14.72)	χ_2_ = 3.669	0.055
Liver disease, yes; n (%)		124 (21.95)	86 (21.39)	38 (23.31)	χ_2_ = 0.25	0.617
COPD, yes; n (%)		24 (4.25)	17 (4.23)	7 (4.29)	χ_2_ = 0.001	0.972
AKI, yes; n (%)		278 (49.2)	164 (40.8)	114 (69.94)	χ_2_ = 39.409	< 0.001
Antibiotics, yes; n (%)		348 (61.59)	230 (57.21)	118 (72.39)	χ_2_ = 11.295	< 0.001
Mechanical ventilation use, yes; n (%)		443 (78.41)	293 (72.89)	150 (92.02)	χ_2_ = 25.092	< 0.001
Vasopressor use, yes; n (%)		154 (27.26)	69 (17.16)	85 (52.15)	χ_2_ = 71.584	< 0.001
RRT, yes; n (%)		49 (8.67)	24 (5.97)	25 (15.34)	χ_2_ = 12.848	< 0.001
Enteral nutrition, yes; n (%)		30 (5.31)	15 (3.73)	15 (9.2)	χ_2_ = 6.905	0.009

Abbreviations: AKI, acute kidney injury; ALB, albumin; ALT, alanine aminotransferase; AST, aspartate aminotransferase; BISAP, bedside index for severity in acute pancreatitis; BUN, blood urea nitrogen; CCI, Charlson comorbidity index; COPD, chronic obstructive pulmonary disease; GCS, Glasgow Coma Scale; HCT, hematocrit; HCT-ALB, difference between HCT and ALB; INR, international normalized ratio; MBP, mean blood pressure; PT, prothrombin time; RDW, red cell distribution width; RRT, renal replacement therapy; SOFA, sequential organ failure assessment; SpO2, oxygen saturation.

### Association between HCT-ALB values and the risk of sepsis

**Figure 2:**
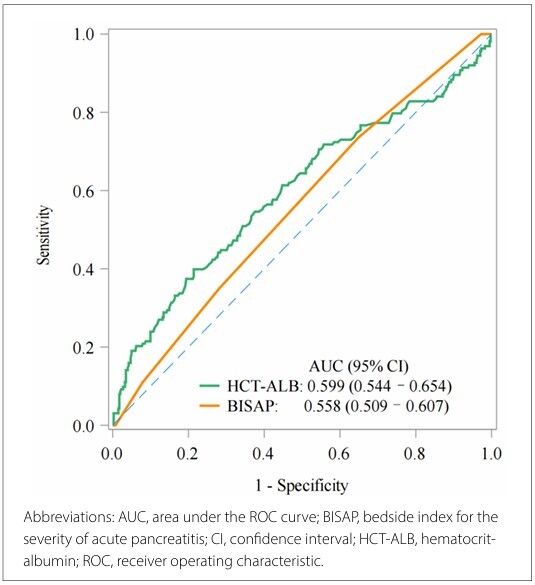
ROC curve analyses of HCT-ALB values and the BISAP score

 As shown in **Appendix 2**, the identified confounding variables encompassed age, sex, ethnicity, marital status, MBP, creatinine, BUN, phosphate, SOFA, BISAP, antibiotic therapy, mechanical ventilation use, vasopressor use, RRT, and enteral nutrition in this analysis (P < 0.05). In the univariate logistic regression model (**Table 2**), HCT-ALB values ≥ 7.6 positively correlated with the risk of sepsis in patients with AP (OR 2.21, 95% CI 1.41–3.49; P < 0.001). In the multivariate logistic regression model, after adjusting for all potential confounding factors (**Table 2**), HCTALB values ≥ 7.6 positively correlated with the risk of sepsis in patients with AP (OR 1.82, 95% CI 1.06–3.14; P = 0.030) compared with a low HCT-ALB (< 0.5) quantile. We also compared the predictive values of HCT-ALB and BISAP using ROC curve analysis. As shown in **Figure 2**, the area under the curve (AUC) value of HCT-ALB in predicting the risk of sepsis in patients with AP was 0.599 (95% CI 0.544–0.654), surpassing the BISAP score (AUC 0.558, 95% CI 0.509–0.607). 

**Table 2:** Relationship between HCT-ALB and the risk of sepsis

**Variables**	**Univariate logistic regression model**		**Multivariate logistic regression model**	
	**OR (95% CI)**	**P value**	**OR (95% CI)**	**P value**
HCT-ALB				
< 0.5	Ref		Ref	
0.5–7.6	1.19 (0.74–1.91)	0.48	1.31 (0.76–2.26)	0.337
≥ 7.6	2.21 (1.41–3.49)	< 0.001	1.82 (1.06–3.14)	0.03

Univariate logistic regression model, not adjusted for confounding variables. Multivariate logistic regression model adjusted for all confounding variables, including age, sex, race/ethnicity, marital status, mean blood pressure, creatinine, blood urea nitrogen, phosphate, sequential organ failure assessment, bedside index for the severity of acute pancreatitis, antibiotics, mechanical ventilation use, vasopressor use, renal replacement therapy, and enteral nutrition. Abbreviations: CI, confidence interval; HCT-ALB, difference between hematocrit and albumin; OR, odds ratio.

### Subgroup analyses

 We performed subgroup analyses based on sex, a history of AKI (no/yes), and SOFA scores (< 2 or ≥ 2) to assess the stability of the association between HCT-ALB values and the risk of sepsis. In **Table 3**, our findings show that male patients with AP and HCT-ALB values ≥ 7.6 in the AKI and SOFA < 2 subgroups had a higher risk of sepsis than those with HCT-ALB values < 0.5, after adjusting for all confounding variables. 

**Table 3:** Subgroup analysis of the association between HCT-ALB and the risk of sepsis

Variables	**Univariate logistic regression model**				**Multivariate logistic regression model**			
	**OR (95% CI)**	**P value**	**OR (95% CI)**	**P value**	**OR (95% CI)**	**P value**	**OR (95% CI)**	**P value**
**Subgroup I: Sex**	**Male**		**Female**		**Male**		**Female**	
*HCT-ALB*								
*< 0.5*	Ref		Ref		Ref		Ref	
*0.5–7.6*	1.11 (0.55–2.21)	0.777	1.25 (0.65–2.42)	0.502	1.62 (0.67–3.9)	0.28	1.40 (0.62–3.18)	0.423
*≥ 7.6*	3.05 (1.64–5.66)	<0.001	1.43 (0.71–2.88)	0.313	3.43 (1.52–7.76)	0.003	1.13 (0.47–2.75)	0.78
**Subgroup II: AKI**	**No**		**Yes**		**No**		**Yes**	
*HCT-ALB*								
*< 0.5*	Ref		Ref		Ref		Ref	
*0.5–7.6*	1.85 (0.85–4.05)	0.121	0.78 (0.42–1.48)	0.454	1.71 (0.68–4.33)	0.257	1.12 (0.54–2.32)	0.77
*≥ 7.6*	2.40 (1.08–5.33)	0.032	1.62 (0.89–2.94)	0.112	1.69 (0.63–4.53)	0.295	2.21 (1.08–4.53)	0.031
**Subgroup III: SOFA**	**SOFA ≥2**		**SOFA <2**		**SOFA ≥2**		**SOFA <2**	
*HCT-ALB*								
*< 0.5*	Ref		Ref		Ref		Ref	
*0.5–7.6*	1.13 (0.63–2.04)	0.688	1.56 (0.65–3.78)	0.323	1.36 (0.68–2.72)	0.377	1.20 (0.4–3.6)	0.749
*≥ 7.6*	1.52 (0.87–2.64)	0.141	4.26 (1.81–10)	< 0.001	1.42 (0.74–2.74)	0.291	4.1(1.33–12.6)	0.014

Univariate logistic regression model, unadjusted confounding variables. Multivariate logistic regression model, Subgroup I: adjusted age, race/ethnicity, marital status, mean blood pressure, creatinine, blood urea nitrogen, phosphate, sequential organ failure assessment, bedside index for severity of acute pancreatitis, antibiotics, mechanical ventilation use, vasopressor use, renal replacement therapy, and enteral nutrition; Subgroup II: adjusted for age, sex, race/ethnicity, marital status, mean blood pressure, creatinine, blood urea nitrogen, phosphate, sequential organ failure assessment, bedside index for the severity of acute pancreatitis, antibiotics, mechanical ventilation use, vasopressor use, renal replacement therapy, and enteral nutrition; Subgroup III: adjusted for age, sex, race/ethnicity, marital status, mean blood pressure, creatinine, blood urea nitrogen, phosphate, bedside index for the severity of acute pancreatitis, antibiotics, mechanical ventilation use, vasopressor use, renal replacement therapy, and enteral nutrition. Abbreviations: HCT-ALB, difference between hematocrit and albumin; OR, odds ratio; CI, confidence interval; AKI, acute kidney injury; SOFA, sequential organ failure assessment.

## DISCUSSION

 This is the first study to investigate the association between HCTALB values and the risk of sepsis in patients diagnosed with AP. We observed an association between HCT-ALB values ≥7.6 and an increased risk of sepsis in patients with AP. This association was particularly robust in relation to men and those in the AKI and SOFA < 2 subgroups. Our data supports the clinical utility of HCT-ALB as a biomarker to assess the risk of sepsis in patients with AP. 

 Recently, with the advancement of research, some biological markers have gained extensive utilization in the clinical prognosis of diseases, such as HCT and ALB.^16-18^ ALB is synthesized in the liver. Serum ALB levels have frequently been reported to be associated with prognosis in hospitalized older adult patients,^19^ as well as in patients with malnutrition,^20^ heart failure,^21^ and sepsis.^19^ ALB exerts various physiological effects, encompassing the regulation of osmotic pressure, safeguarding the microvascular system and mitigating heightened vascular permeability.^22^ It also exhibits antioxidant properties by scavenging free radicals, possesses anticoagulant effects, contributes to maintaining acid-base equilibrium, and demonstrates anti-inflammatory characteristics.^23^ The prognostic value of a single serum ALB measurement, however, may be limited owing to its susceptibility to influence from chronic diseases, nutritional support, and inflammation. A combination of ALB and other indicators may yield more accurate prognostic predictions than the use of ALB alone. In 2022, Liu et al. reported that the lactate-to-albumin (LAR) ratio showed higher accuracy than ALB or lactate alone for all-cause mortality among patients with AP.^24^ HCT-ALB values have recently been investigated as potential indicators. The HCT-ALB value is defined as the difference between the HCT and ALB levels. Previous studies have reported the prognostic significance of HCT-ALB in older adults with sepsis in relation to in-hospital mortality,^11^ and have also reported elevated HCT-ALB values in patients with infectious diseases, thereby establishing it as a robust diagnostic indicator for such conditions.^12^ Therefore, we hypothesized that the HCTALB value might also be a potential biomarker for sepsis risk in patients with AP. 

 After adjusting for age, sex, ethnicity, marital status, MBP, creatinine, BUN, phosphate, SOFA, BISAP, antibiotic therapy, mechanical ventilation use, vasopressor use, RRT, and enteral nutrition, we observed that, compared with a low HCT-ALB (< 0.5) quantile, a high HCT-ALB value (≥ 7.6) was associated with an increased risk of sepsis in patients with AP. The BISAP score, developed in 2008, can be easily calculated using data points available within the first 24 h of presentation to the emergency department, including BUN levels, an abnormal mental status, evidence of SIRS, age ≥ 60 years, and the presence of pleural effusion.^25^ It has been reported that the BISAP score can be used in the early prediction of severity and mortality in AP.^26,27^ In this analysis, we compared the predictive value of HCT-ALB with the BISAP score in predicting the risk of sepsis in patients with AP. Our findings indicated that HCT-ALB might potentially serve as a prognostic indicator, enhancing clinicians’ ability to predict sepsis risk among patients with AP and leading to improved management strategies. In the subgroup analysis, an association between HCT-ALB and the risk of sepsis was observed among men with AP, and those who had AKI or a SOFA score < 2. HCTALB may be more suitable for predicting sepsis risk among men with AP, those with a history of AKI, or those with a SOFA score of < 2. Further investigations are required to investigate the precise mechanism underlying this discovery in future research. 

 This study had some limitations. Concerning patients with multiple ICU admissions, only data concerning the first ICU admission were analyzed, which may have generated a selection bias. This retrospective cohort study used data derived from the MIMIC-IV database. Despite efforts to adjust for confounding variables, it is possible that there were unidentified potential confounders. Moreover, serum ALB or HCT levels may vary over time; however, we only focused on the initial HCT-ALB values without monitoring their dynamic fluctuations. Further validation of the prognostic value of HCT-ALB in patients with AP through prospective multicenter studies is warranted, along with exploration of its underlying mechanisms. 

## CONCLUSION

 High HCT-ALB values ≥ 7.6 were associated with an increased risk of sepsis in patients with AP. Determining the HCT-ALB value may contribute to identifying the risk of sepsis in such patients, thereby improving risk assessment and guiding subsequent interventions. 

## Appendix

### Appendix 1

**Table:** Sensitivity analysis of data pre- and post-interpolation

**Variables**		**Pre-interpolation**	**Post-interpolation**	**Statistics**	**P-value**
MBP, mmHg, Mean ± SD		91.79 ± 19.17	91.71 ± 19.17	t = 0.07	0.943
Respiratory rate, Mean ± SD		21.58 ± 6.6	21.66 ± 6.67	t = −0.18	0.856
Temperature, ^°^C, Mean ± SD		36.84 ± 1	36.84 ± 1	t = 0.01	0.991
BUN, mg/dL, M (Q_1_, Q_3_)		20 (12–37)	20 (12–37)	Z = 0.022	0.983
Creatinine, mg/dL, M (Q _1_, Q_3_)		1.1 (0.7–2)	1.1 (0.7–2)	Z = 0.003	0.997
RDW, %, Mean ± SD		15.21 ± 2.18	15.21 ± 2.18	t = −0.01	0.991
Bilirubin, mg/dL, M (Q _1_, Q_3_)		1.1 (0.6–3)	1.1 (0.6–3)	Z = 0.004	0.997
Bicarbonate, mEq/L, Mean ± SD		20.67 ± 5.65	20.65 ± 5.66	t = 0.05	0.959
Sodium, mEq/L, Mean ± SD		138.39 ± 5.9	138.38 ± 5.91	t = 0.03	0.974
Chloride, mEq/L, Mean ± SD		104.66 ± 7.33	104.65 ± 7.33	t = 0.02	0.982
Phosphate, mg/dL, M (Q _1_, Q_3_)		3.3 (2.3–4.3)	3.3 (2.3–4.2)	Z = 0.002	0.998
Glucose, mg/dL, M (Q _1_, Q_3_)		124.5 (100–174)	124 (100–174)	Z = 0.01	0.992
ALT, IU/L, M (Q_1_, Q_3_)		55 (25, 168)	54 (25–168)	Z = 0.11	0.913
AST, IU/L, M (Q_1_, Q_3_)		79.5 (38–187.5)	78 (38–187)	Z = 0.151	0.88
SpO_2_, %, Mean ± SD		95.92 ± 4.58	95.93 ± 4.58	t = −0.03	0.979
INR, M (Q_1_, Q_3_)		1.3 (1.2–1.6)	1.3 (1.1–1.6)	Z = 1.348	0.177
PT, sec, M (Q_1_, Q_3_)		14.5 (13–17.5)	14.4 (13–17.3)	Z = 0.339	0.735
AKI, n (%)					
	*No*	285 (50.62)	287 (50.8)	χ^2^ = 0.003	0.953
	*Yes*	278 (49.38)	278 (49.2)		
SOFA, M (Q_1_, Q_3_)		2 (0–5)	2 (0–5)	Z = −0.035	0.972
GCS, mean ± SD		14.53 ± 1.58	14.53 ± 1.58	t = −0.02	0.986

Abbreviations: MBP, mean blood pressure; BUN, blood urea nitrogen; RDW, red cell distribution width; ALT, alanine aminotransferase; AST, aspartate aminotransferase; SpO_2_, oxygen saturation; INR, international normalized ratio; PT, prothrombin time; AKI, acute kidney injury; SOFA, sequential organ failure assessment; GCS, Glasgow Coma Scale; SD, standard deviation.

### Appendix 2

**Table:** Screening of the confounding variables

**Variables**		**OR (95% CI)**	**P value**
Age		0.99 (0.98–1)	0.105
Sex			
	*Women*	Ref	
	*Men*	0.93 (0.65–1.34)	0.704
Marital status			
	*Married*	Ref	
	*Never married*	1.26 (0.82–1.93)	0.299
	*Divorced*	0.89 (0.42–1.88)	0.765
	*Widowed*	0.68 (0.33–1.41)	0.298
	*Unknown*	1.16 (0.64–2.1)	0.623
Ethnicity			
	*African American*	Ref	
	*European*	1.1 (0.57–2.11)	0.775
	*Other*	1.53 (0.7–3.37)	0.29
	*Not known*	1.42 (0.67–2.98)	0.356
MBP		0.99 (0.98–0.99)	0.003
Respiratory rate		1 (0.98–1.03)	0.739
Temperature		1.21 (1–1.47)	0.056
Creatinine		1.15 (1.05–1.26)	0.004
Hemoglobin		1.01 (0.93–1.09)	0.842
BUN		1.01 (1.01–1.02)	0.002
RDW		1.05 (0.97–1.14)	0.254
Antibiotics			
	*No*	Ref	
	*Yes*	1.96 (1.32–2.91)	< 0.001
Platelet		1 (1–1)	0.454
Bilirubin		1.01 (0.98–1.05)	0.389
Bicarbonate		0.95 (0.92–0.99)	0.005
Sodium		1.01 (0.98–1.04)	0.65
Potassium		1.16 (0.96–1.41)	0.132
Chloride		1.02 (1.00–1.05)	0.084
Phosphate		1.16 (1.05–1.27)	0.002
Glucose		1 (1–1)	0.08
ALT		1 (1–1)	0.469
AST		1 (1–1)	0.191
SpO_2_		0.99 (0.95–1.03)	0.5
INR		1.18 (0.99–1.41)	0.069
PT		1.01 (1–1.03)	0.107
Lipase			
	*< 208 U/L*	Ref	
	*≥ 208 U/L*	0.84 (0.54–1.32)	0.459
	*Unknown*	0.81 (0.51–1.29)	0.383
SOFA		1.19 (1.12–1.26)	< 0.001
BISAP		1.23 (1.03–1.48)	0.025
GCS		0.96 (0.86–1.07)	0.438
CCI		1.01 (0.94–1.1)	0.728
Heart failure			
	*No*	Ref	
	*Yes*	1.07 (0.65–1.79)	0.782
Hypertension			
	*No*	Ref	
	*Yes*	0.82 (0.57–1.19)	0.294
Diabetes			
	*No*	Ref	
	*Yes*	0.78 (0.52–1.17)	0.222
Renal failure			
	*No*	Ref	
	*Yes*	1.70 (0.98–2.95)	0.057
Liver disease			
	*No*	Ref	
	*Yes*	1.12 (0.72–1.72)	0.618
COPD			
	*No*	Ref	
	*Yes*	1.02 (0.41–2.5)	0.972
AKI			
	*No*	Ref	
	*Yes*	3.38 (2.29–4.98)	< 0.001
Mechanical ventilation use			
	*No*	Ref	
	*Yes*	4.29 (2.34–7.88)	< 0.001
Vasopressor use			
	*No*	Ref	
	*Yes*	5.26 (3.52–7.86)	< 0.001
RRT			
	*No*	Ref	
	*Yes*	2.85 (1.58–5.16)	< 0.001
Enteral nutrition			
	*No*	Ref	
	*Yes*	2.61 (1.25–5.48)	0.011

Abbreviations: AKI, acute kidney injury; ALT, alanine aminotransferase; AST, aspartate aminotransferase; BISAP, bedside index for severity in acute pancreatitis; BUN, blood urea nitrogen; CI, confidence interval; CCI, Charlson comorbidity index; COPD, chronic obstructive pulmonary disease; GCS, Glasgow Coma Scale; INR, international normalized ratio; MBP, mean blood pressure; OR, odds ratio; PT, prothrombin time; RDW, red cell distribution width; SOFA, sequential organ failure assessment; RRT, renal replacement therapy; SpO_2_, oxygen saturation.
